# Accuracy and Safety of Computer-Assisted Surgery (CAS) in the Treatment of TMJ Ankylosis—Report of Several Cases and Review of the Literature

**DOI:** 10.3390/cmtr19010016

**Published:** 2026-03-19

**Authors:** Andrei Krasovsky, Boaz Frenkel, Michal Even Almos, Yair Israel, Dekel Shilo, Amir Bilder, Tal Capucha, Omri Emodi

**Affiliations:** 1 Department of Oral and Maxillofacial Surgery, Rambam Medical Care Campus, Haifa 9602, Israel; dr.boazfrenkel@gmail.com (B.F.);; 2 Ruth & Bruce Rappaport Faculty of Medicine, Technion-Israel Institute of Technology, Haifa 9602, Israel

**Keywords:** TMJ ankylosis, VSP, 3D printing, cutting guides, navigation, complications

## Abstract

Background: Temporomandibular joint (TMJ) ankylosis is an uncommon condition in the modern world, yet it remains a significant treatment challenge. One of the main intraoperative difficulties is accurately and safely resecting the ankylotic mass. Objective: This study seeks to share our clinical experience with various types of complications and to review the literature on the clinical and technological evidence regarding the accuracy of surgical detachment of the ankylotic mass from the skull. Methods: A literature review was conducted using PRISMA (Preferred Reporting Items for Systematic Review and Meta-Analysis) guidelines. Search strategies were categorized into search 1 for evaluating 3D-printed technology and search 2 for computer-assisted surgical navigation. Results: One study was selected for search 1 and 2 for search 2. Also, three cases of intraoperative surgical complications associated with the resection of the ankylotic mass were presented. The 3D surgical cutting guides were found to be accurate in guiding the superior, inferior, and depth of the osteotomy. Angulation control was less than optimal. Navigation guiding proved accurate in maintaining the planned thickness of the skull base and the anterior wall of the external auditory canal. Conclusion: Navigation guiding is a superior method for achieving predictable anatomical resection of the ankylotic mass.

## 1. Introduction

Temporomandibular joint (TMJ) ankylosis is most often an acquired condition characterized by partial or complete fibrous, fibro-osseous, or completely osseous fusion of the mandibular condyle with the skull base, usually in the younger population [1]. Trauma is the most common cause of TMJ ankylosis in developed countries, while infection is the leading cause in the third world [2]. Surgical options for treating TMJ ankylosis include gap arthroplasty, interpositional arthroplasty, and total joint reconstruction (TJR) [3]. Total joint reconstruction can be classified as autogenous replacement, such as costochondral (CCG) and sternoclavicular grafts (SCG); microvascular reconstruction; or alloplastic replacement. Autogenous options are usually preferred in young patients [2]. Each surgical option begins with resection of the ankylotic mass. The initial stage can be extremely challenging because it requires precise, complete excision of the ankylotic mass. A preauricular incision usually provides good exposure of the lateral and anterior boundaries of the mass. However, visualizing the posterior and medial borders of the mass can be very difficult and deceptive, risking damage to nearby structures, such as the ear canal, skull base perforations, or the maxillary artery [4,5]. Computer-Assisted Surgery (CAS) modalities, including virtual surgical planning (VSP), preoperative computer/model-based simulation [6,7], patient-specific 3D-printed surgical cutting guides [8], and surgical navigation [9], have been documented to improve the accuracy and safety of ankylotic mass excision.

In this work, we present our experience with various surgical complications arising from operative inaccuracy during detachment of the ankylotic mass from the skull base. Subsequently, a review of the literature evaluating the accuracy of CAS for the excision of ankylotic masses is provided and discussed. This work aims to highlight surgical pitfalls during the most critical phase of TMJ ankylosis surgery and to provide evidence-based insights to enhance precision and prevent these issues.

## 2. Materials and Methods

Two searches were conducted, the first (search 1) focusing on the application of 3D-printed devices in TMJ ankylosis surgery and the second (search 2) on the application of surgical navigation systems in TMJ ankylosis surgery. Publications were screened to evaluate the postoperative accuracy of the preplanned osteotomy for resecting the ankylotic mass and the types of osteotomy-related complications. No formal protocol was registered for this study prior to its commencement.

### 2.1. Search Strategy

The methodology was based on the PRISMA (Preferred Reporting Items for Systematic Review and Meta-Analysis) guidelines [10]. The following databases were searched for relevant literature published between January 1990 and December 2025: PubMed, Scopus, and Web of Science. For PubMed, Cochrane-style Medical Subject Headings (MeSH) were used. The MeSH terms in search 1 included: TMJ ankylosis, cutting guide, and 3D printing. The MeSH terms in search 2 included: TMJ ankylosis and navigation.

### 2.2. Eligibility Criteria

The PEO (population, exposure, and outcome) framework was used:

- Population: individuals diagnosed with TMJ ankylosis.
- Exposure: surgical treatment using 3D-printed cutting guides or navigation.
- Outcome: postoperative evaluation of the osteotomy accuracy and osteotomy-related complications.

English-only publications were reviewed, and selected studies were randomized controlled trials (RCTs), comparative trials, prospective or retrospective studies, case series, and case reports. Initially, titles and abstracts were reviewed to identify suitable articles, and two authors further evaluated the selected manuscripts.

Based on title and abstract relevance screening, animal studies and narrative or systematic reviews were excluded. Reviewed full-text publications were considered ineligible if they did not clearly describe the relevant outcomes. Studies were excluded from search 1 if they did not use 3D-printed surgical cutting guides and from search 2 if they did not use navigation.

### 2.3. Study Selection and Data Extraction

Each study was evaluated separately by two investigators for the study design, type of surgical intervention, type of VSP method used, 3D-printed models and cutting guides, and surgical outcomes. Two primary outcomes of interest were postoperative evaluation of preplanned osteotomy accuracy and osteotomy-related complications.

### 2.4. Quality Assessment

To assess the risk of bias in studies, the Joanna Briggs Institute (JBI) Manual for Evidence Synthesis 2024 edition was used [11]. The selected articles were assessed and subgrouped into high risk of bias (JBI score ≤ 49%), moderate risk of bias (JBI score 50–69%), and low risk of bias (JBI score > 70%) [12]. The Oxford Level of Evidence was used to assess the strength of the studies [13].

## 3. Results

Concerning our clinic’s experience, we present three cases with different types of osteotomy errors specifically related to the surgical separation of the ankylotic mass from the base of the skull.

### 3.1. Case 1

**Figure 1:**
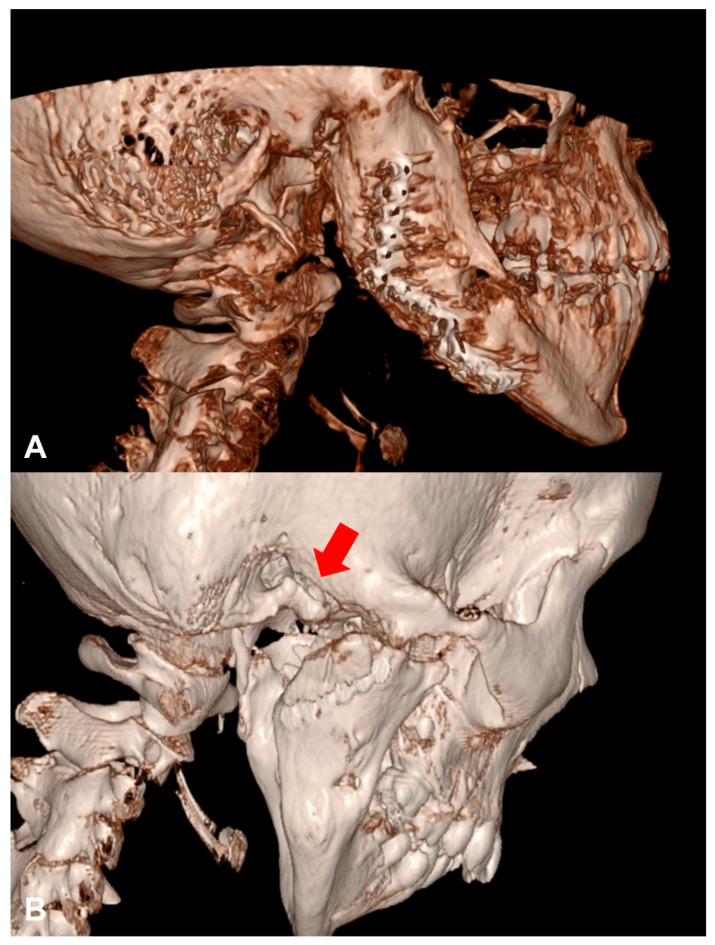
(**A**) Preoperative CBCT 3D reconstruction demonstrating full bony ankylosis of the right TMJ. (**B**) Postoperative CT 3D reconstruction demonstrating right bony external acoustic meatus perforation (red arrow).

A 10-year-old female with bilateral full bony TMJ ankylosis as a complication of distraction osteogenesis for mandibular advancement underwent bilateral gap arthroplasty. The unusual fusion area of the right bony ankylosis was located more anteriorly at the eminence, while the glenoid fossa was barely affected by the ankylosis (Figure 1A). Due to the exceptionally anterior position of the ankylotic mass, the use of 3D cutting guides was avoided because the surgical exposure required to insert the guides properly was too extensive. The craniomaxillofacial (CMF) navigation system is not yet available at our medical center. During the resection of the right ankylotic mass, a perforation of the bony external acoustic meatus occurred (Figure 1B). Initially, the patient was treated with antibiotic eardrops applied to a wick and later underwent tympanoplasty.

### 3.2. Case 2

**Figure 2:**
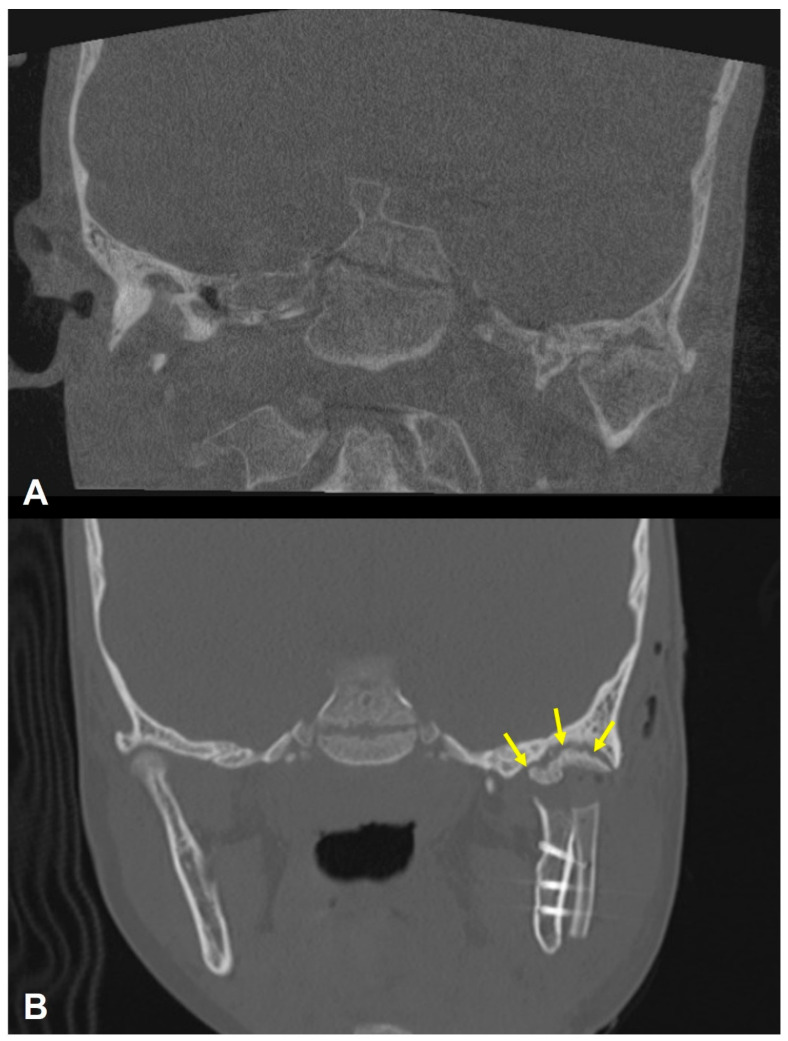
(**A**) Preoperative coronal CBCT demonstrating fibro-osseous fusion of the left condyle with the skull base. (**B**) Postoperative coronal CT demonstrating a partially removed ankylotic mass (yellow arrows).

A 7-year-old male with left fibro-osseous TMJ ankylosis (Figure 2A), suspected to result from early trauma, underwent excision of the of the ankylotic mass with CCG reconstruction. Virtual surgical planning using Geomagic Freeform software (3D Systems, Rock Hill, SC, USA), 3D printing of anatomical models of the ankylotic joint with Hyper Speed PLA filament (Raise3D Technologies, Bellevue, WA, USA), and a patient-specific 3D surgical cutting guide directing the subcondylar osteotomy line fabricated using BioMed Surgical Guide Resin (Formlabs Inc., Somerville, MA, USA), were used during surgery. A surgical guide for osteotomy line detaching the ankylotic mass from the skull base was not planned. Postoperative Computed Tomography (CT) revealed a superior remaining ankylotic part attached to the roof of the glenoid fossa (Figure 2B). Postoperative recovery was uneventful, with a maximal interincisal opening (MIO) of 35 mm during physical therapy, which began 10 days after surgery, in accordance with the Kaban protocol [14].

### 3.3. Case 3

**Figure 3:**
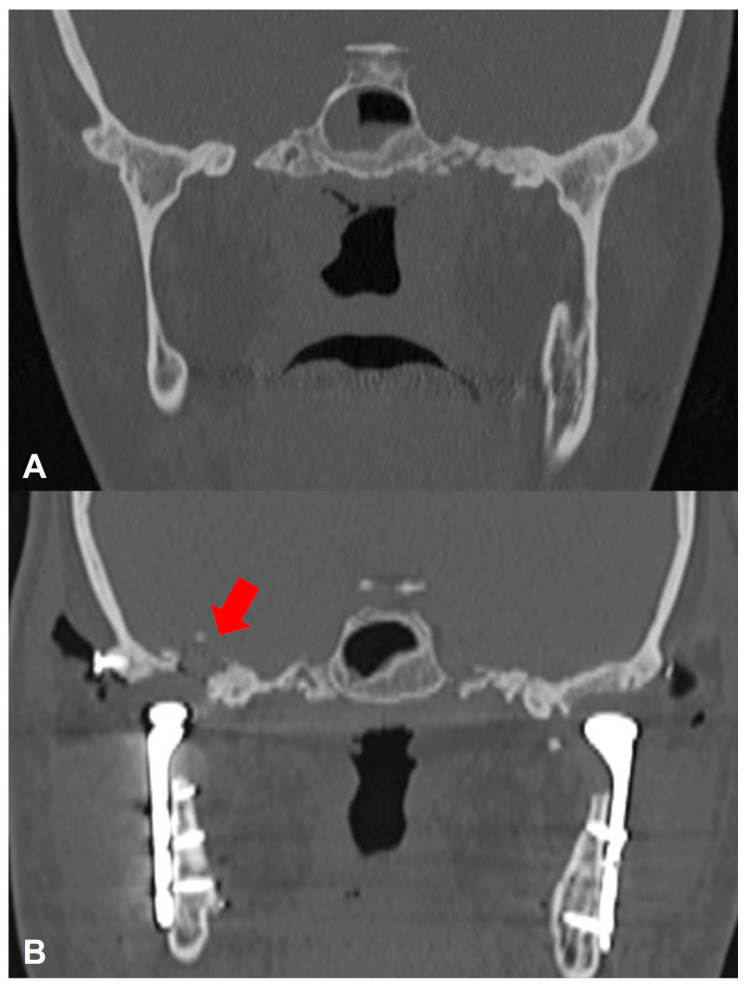
(**A**) Preoperative axial CT demonstrating bilateral full bony ankylosis. (**B**) Postoperative axial CT demonstrating skull base perforation at the right glenoid fossa (red arrow).

A 26-year-old female with bilateral full bony ankylosis as an untoward sequela to previous high condylectomy, diskectomy, and coronoidectomy (Figure 3A) had bilateral TJR surgery using stock Zimmer-Biomet TMJ devices (Biomet Microfixation Inc., Jacksonville, FL, USA). Based on preoperative VSP with Geomagic Freeform software (3D Systems, Rock Hill, SC, USA), an anatomical model of the mandible fused to the skull base was printed using Hyper Speed PLA filament (Raise3D Technologies, Bellevue, WA, USA), and surgical simulation of the osteotomy was performed by the leading surgeon, using patient-specific 3D printed cutting guides fabricated using BioMed Surgical Guide Resin (Formlabs Inc., Somerville, MA, USA), after which the fossa and condyle trials were attached. During the surgery, resecting the medial and superior borders of the right ankylotic mass posed a significant challenge in defining the anatomical boundaries. An intraoperative CT scan was performed to validate the temporary bilateral fixation of the glenoid fossa and condyle trials after the resection of ankylosis and the completion of any additional osteotomies required for optimal fit. Perforation into the middle cranial fossa was identified on the right side (Figure 3B). Postoperative recovery was uneventful, with an MIO of 30 mm. Neurosurgery consultation recommended a short course of prophylactic antibiotics and anticonvulsants.

A summary of the three clinical cases, including complications and management, is presented in Table 1.

#### 3.3.1. Search 1

**Figure 4:**
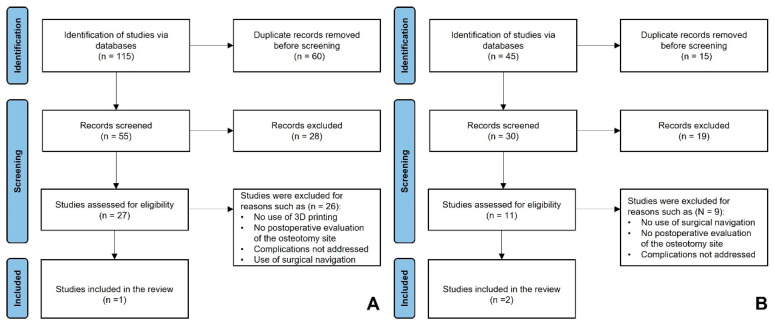
(**A**) Search 1 PRISMA flowchart of the reviewed studies. (**B**) Search 2 PRISMA flowchart of the reviewed studies.

A total of 115 records were identified through electronic databases. After removing 60 duplicates, 55 records remained for title and abstract screening. Of these, 28 were excluded based on relevance. Subsequently, 27 full-text articles were retrieved and assessed for eligibility. Of these, 26 were excluded for reasons such as no use of 3D printing, no postoperative evaluation of the osteotomy site, unaddressed complications, and use of surgical navigation. A summary of the study selection process is presented in Figure 4A.

#### 3.3.2. Search 2

A total of 45 records were identified through electronic databases. After removing 15 duplicates, 30 records remained for title and abstract screening. Of these, 19 were excluded based on relevance. Subsequently, 11 full-text articles were assessed for eligibility, out of which nine were excluded for reasons such as no use of surgical navigation, no postoperative evaluation of the osteotomy site, and unaddressed complications. A summary of the study selection process is presented in Figure 4B.

In total, three studies were included in the review. The characteristics of the reviewed studies are presented in Table 2. Outcomes, quality, and the level of evidence for the studies are summarized in Table 3.

## 4. Discussion

The incidence of TMJ ankylosis in developed countries is low due to advances in the management of condylar fractures and infective conditions [18]. However, the management of the condition, particularly in growing patients, remained challenging and had long-term consequences, requiring a multidisciplinary approach [19].

The main objectives in managing TMJ ankylosis are the removal of ankylotic tissue and the restoration of joint function and normal physiology. Surprisingly, while there is plenty of literature discussing the second objective, there is very scarce evidence addressing the accuracy of the first. Accurate resection of the ankylotic mass is crucial to avoid the previously described intraoperative complications. It is also essential for the predictability of the following step of restoring joint function.

In alloplastic patient-specific TJR procedures, a 3D-printed cutting guide facilitates flat resection of the ankylotic mass and accurate screw hole placement to ensure correct positioning of the glenoid fossa component [20]. In stock alloplastic TJR cases, 3D-printed anatomical models can be used for preoperative surgical simulation to verify the virtual plan [21]. A major drawback of patient-specific 3D-printed cutting guides for detaching the ankylotic mass from the skull base is the difficulty in predicting the safe medio-lateral depth and preventing cranial injury [22].

When autogenous reconstruction is planned, the benefit of flat-gap osteoplasty using a 3D-printed cutting guide is uncertain. A truly curved glenoid fossa is preferred to facilitate neocondyle remodeling and adaptation, thereby improving long-term functional results [23]. However, guided, precise volumetric curved-form creation is not feasible when relying solely on 3D templates.

For this purpose, surgical navigation technology can offer a plausible solution. The navigation wand provides intermittent real-time orientation feedback during osteotomy advancement, especially when approaching danger zones. Moreover, the active end of the cutting bur or ultrasonic saw can, by itself, be calibrated to serve as a navigation tool, providing continuous real-time feedback during the osteotomy phase [24,25]. A navigation system can also serve to facilitate the proper positioning of the cutting guide [26,27]; however, contouring a round glenoid fossa will not be achieved.

Intraoperative CT was also documented to be used during TMJ ankylosis surgeries [21]. The added value of this entity is primarily in validating the proper positioning of the fossa and condyle prostheses in alloplastic TJR cases. Guiding the osteotomy process using intraoperative CT is less practical.

Although a considerable number of reports described CAS implementation to facilitate detachment of the ankylotic mass from the skull base, this literature review identified only three articles that met the inclusion criteria for numerically measuring the osteotomy accuracy of this step [15,16,17]. Only gap arthroplasty procedures were analyzed.

Mounir et al. examined osteotomy accuracy in five patients using patient-specific polylactic acid (PLA)-printed cutting guides [15]. The guides included two slits to determine the superior and inferior limits of the planned bony gap. The depth of the osteotomy was measured from the outer surface of the slit to 2 mm shorter than the medial extension of the ankylotic mass. Drills, marked to the desired depth of penetration, were introduced first and followed by the fine fissure burr osteotomies. Small osteotomies tapped the remaining eggshell. No significant difference was found between the virtually planned bony gap distance and the postoperative bony gap distance measured on CT. However, the drilling angulation close to the depth of the bony mass was less than ideal. Based on our experience, it is not feasible to guide the angulation of a drill or saw using a PLA guide without metal sleeves or slots. To achieve proper angulation, the active tool must rest on at least one side of the guide. However, the rotating or oscillating motion tends to cut through and deform the PLA material, which undermines the guidance for angulation. Creating robust, wide guiding shoulders for the cutting guide to address this challenge is impractical for such delicate osteotomies. Moreover, larger cutting guides will automatically require greater soft-tissue exposure and higher retraction forces, elevating the risk of temporal branch weakness, as documented in two patients in this work. One possible solution is to use a metal 3D-printed cutting guide, although this will dramatically increase the cost and make it less available for in-house manufacturing. As presented in case 3, even small errors in cutting depth and angulation may lead to cranial base perforation. As already mentioned, using a cutting guide can only perform a flat osteotomy and has limited ability to remove the most superior ankylotic mass, as depicted in case 2.

Navigation accuracy performance was similarly tested in two different studies. He et al. retrospectively measured the residual bone thickness in the skull bone and the anterior wall of the external auditory canal [16]. Eighteen patients were treated using computer-assisted navigation and compared with 19 patients treated without it. An intraoperative navigation probe was used intermittently to monitor the safety distance of 3 mm from the middle cranial fossa and bony external auditory canal, allowing careful shaping of the glenoid fossa with a round bar. On CT, postoperative skull base thickness was significantly greater in the non-navigation group, nearly twice as large, indicating that less ankylotic mass was removed. The difference in the anterior wall of the auditory canal was not significant between groups. This might be due to the auditory canal being visualized as an orientation cue, while no such cues are available for the position of the skull base. Yu et al. evaluated the image-guided navigation system by reporting data from four patients and comparing postoperative skull base thickness on the operated side with that on the contralateral side [17]. No significant difference was found between the two sides, meaning that the normal anatomical boundary between the cranial fossa and the glenoid fossa was restored. In this work, the round-bur drill tip was calibrated and positioned in real time on the screen, in contrast to the occasional navigation-probe application by He et al.

The main disadvantage of using a navigation system is its high cost, which limits accessibility for many medical centers. Another documented issue is a potential surface registration systemic error of approximately 1.5 mm, which sets a minimal safety margin for resection [16].

In complex anatomical cases, which can lead to loss of orientation, as shown in case 1, the risk can be easily avoided by using a navigation system. At the same time, we regard this case as inappropriate for cutting guides.

The literature documents various postoperative complications in TMJ ankylosis treatment, including facial nerve weakness, infection, anterior open bite, unstable occlusion, ear perforation, cerebrospinal fluid (CSF) leakage through the ear, salivary fistula, Frey’s syndrome, recurrence, and heterotrophic bone formation [4,28,29,30]. When facial nerve injuries are attributed to excessive intraoperative retraction forces, ear and cranial fossa perforation and incomplete ankylotic mass removal can be attributed to intraoperative loss of orientation in the very limited surgical field. In the cases presented by our department, no surgical re-entry was considered solely on the basis of radiologically observed complications. Postoperative functional assessment and follow-ups were the guiding criteria. In the case of partial removal of an ankylotic mass, since the functional outcome was satisfactory, a standard physical therapy protocol was used without modification. A case of cranial fossa perforation was immediately referred for consultation by a neurosurgeon. Although postoperative neurological signs and symptoms were not evident, an open fracture protocol was implemented and included 1 g of Cefazolin three times a day and 240 mg of Gentamicin once a day, all for a total of 3 days. Also, Levetiracetam at 1 g per day was prescribed until follow-up in 3 weeks, with a brain CT. Finally, in the case of bony external auditory canal perforation, the primary concern was CSF leakage. After that was excluded, an otolaryngologist recommended a two-week course of Ciprofloxacin ear drops, three times a day, with subsequent follow-up and maintenance of dry ear precautions. According to the literature, tympanoplasty is recommended if the perforation does not close spontaneously within 3 to 6 months and there is no secondary infection [31]. Since no spontaneous closure was observed after 6 months, a tympanoplasty was recommended for the patient.

## 5. Conclusions

Based on our clinical experience and the literature review, guided surgery should be the standard approach for treating TMJ ankylosis to ensure precision and safety. When an alloplastic TJR is not indicated, which is often the case in growing patients, a navigation system is the preferred guiding technology. Using navigation control can help achieve a more anatomical glenoid fossa shape, thereby enhancing the joint’s ability to remodel during growth.

## Figures and Tables

**Table 1:** Summary of clinical cases.

	Gender (M/F)	Age (Years)	Type Ankylosis	Side	Type of Intervention	Radiological PO Complication	Clinical PO Manifestation	Management
Case 1	F	10	Full bony	Bilateral	Gap arthroplasty	Perforation of the bony external acoustic meatus on the right side	Partial hearing loss on the right side. MIO—35 mm	Tympanoplasty
Case 2	M	7	Fibro-osseous	Left	CCG	Partial removal of ankylotic mass	Uneventful, MIO—35 mm	PT 10 days after surgery
Case 3	F	26	Full bony	Bilateral	TJR	Perforation of the middle cranial fossa on the right side	Uneventful, MIO—30 mm	Prophylactic antibiotics and anticonvulsants

PO—postoperative; CCG—costochondral graft; TJR—total joint reconstruction; MIO—maximal interincisal opening, PT—physical therapy.

**Table 2:** Characteristics of the reviewed studies.

	Study	Gender (M/F)	Sample Size (Age Range/Mean)	Pathology	Intervention Type	CAS Type
Search 1						
	Mounir et al., 2020 [15]	3/2	5 (11–30 years, mean 19.6 years)	Unilateral TMJ ankylosis	Gap arthroplasty	3D-printed PLA cutting guides
Search 2						
	He et al., 2017 [16]	20/17	37 (6–62 years, mean 24.8 years)	Uni/bilateral TMJ ankylosis	Gap arthroplasty	Navigation
	Yu et al., 2009 [17]	2/2	4 (11–17 years, mean 14.25 years)	Unilateral TMJ ankylosis	Gap arthroplasty	Navigation

CAS—Computer-Assisted Surgery; PLA—polylactic acid.

**Table 3:** Study outcomes, quality of studies, and level of evidence.

Study	Osteotomy Accuracy Analysis Method	Osteotomy Accuracy Analysis Results	Disadvantages	Complications	Risk of Bias (JBI Checklist)	Level of Evidence
Mounir et al., 2020 [15]	Superimposing preoperative and postoperative CT scans.	Planned virtual bony gap vs. postoperative bony gap—mean (SD) 0.42 (0.444) mm. Not significant difference (t = 2.12, *p* = 0.10) between planned and postoperative gaps.	Suboptimal guidance drilling angulations close to the depth of the bony mass. Adaptation of the cutting guide required wide soft tissue dissection and firm retraction.	Temporary paralysis TFN—*n* = 2 (40%)	Low	Level 4—case series
He et al., 2017 [16]	Postoperative CT to measure residual bone thickness in the skull base and anterior wall of the external auditory canal.	Thickness of the skull base (mm): Navigation—3.86 ± 1.95 Control—6.01 ± 3.07 Thickness of the anterior wall of the external auditory canal (mm): Navigation—3.43 ± 2.40 Control—4.26 ± 2.78 Significant difference between two groups (*p* = 0.009)	Surface registration method of navigation system may be subject to systematic error of 1.5 mm	Ankylosis recurrence: Navigation *n* = 1 (5.6%) Control *n* = 4 (21.1%)	Low	Level 3—retrospective cohort study
Yu et al., 2009 [17]	Postoperative CT to measure thickness of skull base between middle cranial fossa and reconstructed glenoid fossa.	Minimal mean thickness of skull base [mm (S.D.)]: Operated side—1.97 (0.19) Contralateral side—2.10 (0.29) No significant difference between sides (*p* = 0.579)	Presurgical preparation and installation of trackers and sensors followed by registration. Additional staff member required during surgery to manipulate the computer	Non	Low	Level 4—case series

CT—Computed Tomography; TFN—Temporal branch of Facial Nerve; S.D.—Standard Deviation.

## Data Availability

The raw data supporting the conclusions of this study are available from the authors upon reasonable request.
